# Impact of Right Ventricular Trabeculation on Right Ventricular Function in Patients With Left Ventricular Non-compaction Phenotype

**DOI:** 10.3389/fcvm.2022.843952

**Published:** 2022-04-12

**Authors:** Anna Réka Kiss, Zsófia Gregor, Adrián Popovics, Kinga Grebur, Liliána Erzsébet Szabó, Zsófia Dohy, Attila Kovács, Bálint Károly Lakatos, Béla Merkely, Hajnalka Vágó, Andrea Szũcs

**Affiliations:** Heart and Vascular Center of Semmelweis University, Budapest, Hungary

**Keywords:** right ventricle (RV), right ventricular function, cardiac magnetic resonance (CMR), left ventricular non-compaction, non-comaption, trabecula, trabeculation

## Abstract

Right ventricular (RV) involvement in left ventricular (LV) non-compaction (LVNC) remains unknown. We aimed to describe the RV volumetric, functional, and strain characteristics and clinical features of patients with LVNC phenotype and good LV ejection fraction (EF) using cardiac magnetic resonance to characterize RV trabeculation in LVNC and to study the relationships of RV and LV trabeculation with RV volume and function. This retrospective study included 100 Caucasian patients with LVNC phenotype and good LV-EF and 100 age- and sex-matched healthy controls. Patients were further divided into two subgroups according to RV indexed trabecular mass [RV-TMi; patients with RV hypertrabeculation (RV-HT) vs. patients with normal RV trabeculation (RV-NT)]. We measured the LV and RV volumetric, functional, and TMi values using threshold-based postprocessing software and the RV and LV strain values using feature tracking and collected the patients' LVNC-related clinical features. Patients had higher RV volumes, lower RV-EF, and worse RV strain values than controls. A total of 22% of patients had RV-TMi values above the reference range; furthermore, RV-HT patients had higher RV and LV volumes, lower RV- and LV-EF, and worse RV strain values than RV-NT patients. We identified a strong positive correlation between RV- and LV-TMi and between RV-TMi and RV volumes and a significant inverse relationship of both RV- and LV-TMi with RV function. The prevalence of LVNC-related clinical features was similar in the RV-HT and RV-NT groups. These results suggest that some patients with LVNC phenotype might have RV non-compaction with subclinical RV dysfunction and without more severe clinical features.

## Introduction

Left ventricular non-compaction (LVNC) has become a well-known clinical entity, and the amount of research on this topic is increasing. Nevertheless, right ventricular (RV) involvement in LVNC remains a controversial issue with little available data. Compared with those of the left ventricle (LV), the unique shape of the RV and its physiologically greater quantity of endocardial trabeculae make it difficult to evaluate RV hypertrabeculation. Despite the lack of established diagnostic criteria, case reports suggest the existence of isolated RV non-compaction (1–3). The negative relationship between LV trabeculation and LV ejection fraction (EF) has prompted questions about whether RV trabeculation is similarly associated with RV function, which has been shown to be an important prognostic parameter in several cardiac diseases, including LVNC (4–7). The impact of LVNC on RV trabeculation and RV function and the presence of RV dysfunction have not been described. Furthermore, the possibility of RV involvement raises further questions regarding affected patients' morphologic diagnosis, clinical features, incidence, and prognosis.

Our study aimed to describe the RV volumetric, functional and feature-tracking strain parameters of patients with LVNC phenotype and good LV-EF and compare them to those of a healthy control group using cardiac magnetic resonance (CMR); to characterize RV trabeculation in patients with LVNC phenotype; and to describe the relationships of RV and LV trabeculation with RV volume and function. Furthermore, we studied the connection between clinical features and the extent of RV trabeculation in patients with LVNC phenotype.

## Materials and Methods

### Study Population

**Figure 1:**
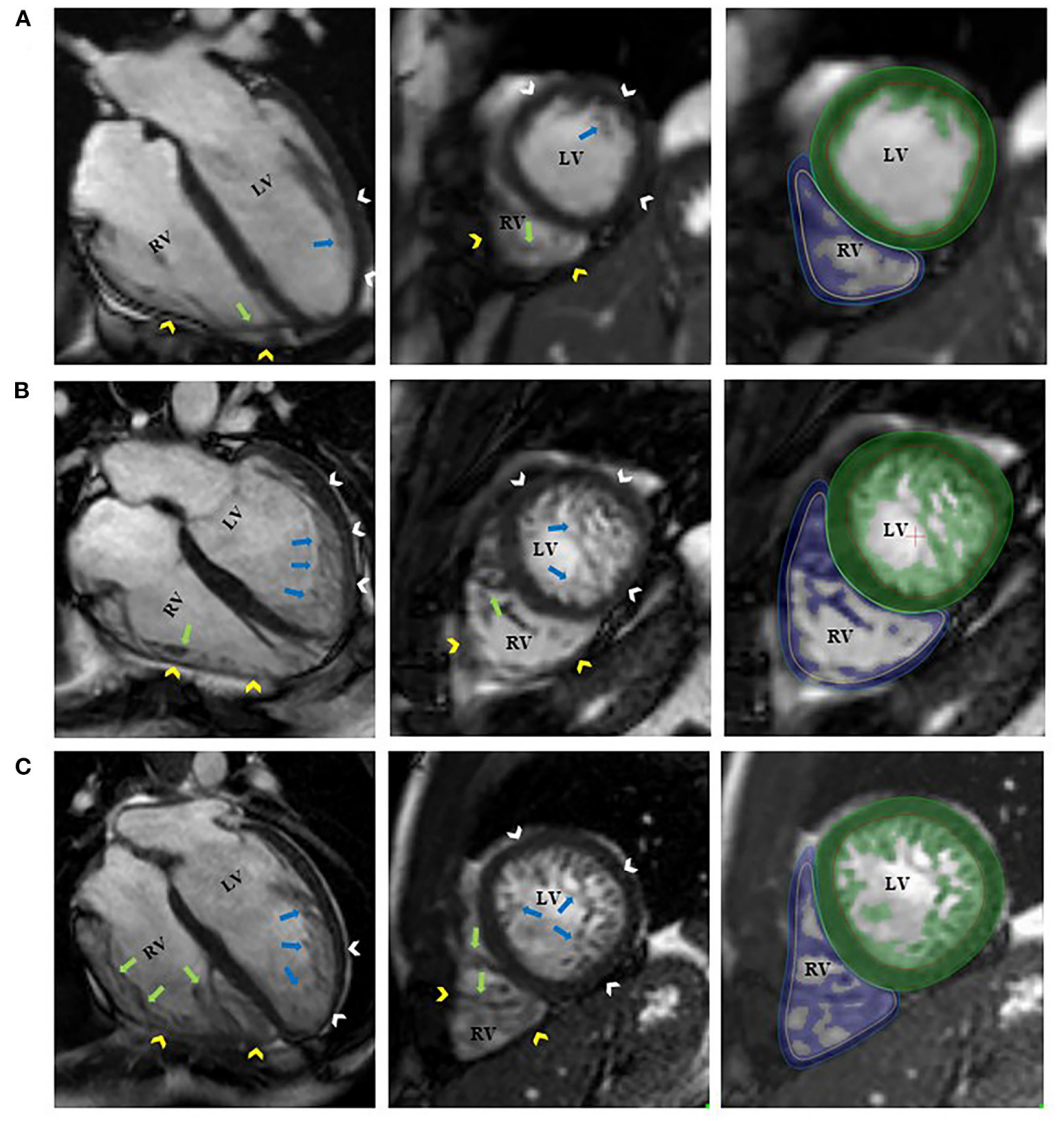
Representative 4-chamber long-axis and short-axis images of a control participant [**(A)**, RV-TMi = 12.6 g/m^2^, age and sex-specific reference: 13.9 (11.9, 19.3) g/m^2^]; a patient with left ventricular non-compaction phenotype and normal amount of right ventricular trabeculation [**(B)**, RV-TMi = 25.2 g/m^2^, age and sex-specific reference: 21.8 ± 5.2 g/m^2^]; and a patient with left ventricular non-compaction phenotype and right ventricular hypertrabeculation [**(C)**, RV-TMi = 32.9 g/m^2^, age and sex-specific reference: 19.9 ± 3.7 g/m^2^]. The white and yellow arrowheads represent the left and right ventricular compacted myocardium. The blue and green arrows represent the left and right ventricular endocardial trabeculation. The green and blue areas represent the myocardial mass, including the endocardial trabeculation of the left and right ventricles, respectively. LV, left ventricle; LVNC, left ventricular non-compaction; RV, right ventricle; RV-TMi, right ventricular trabeculated myocardial mass index.

This retrospective study included 100 Caucasian patients with the LVNC phenotype and 100 age- and sex-matched healthy volunteers (Table 1, Figure 1). CMR examinations were performed at the same institute, and all patients completed a questionnaire for collection of demographic data, cardiovascular symptoms, medical history, medication use, and sports activity. The diagnosis of the LVNC phenotype was established when the CMR-based criteria of both Petersen et al. (non-compacted/compacted ratio > 2.3) and Jaquier et al. (trabeculated LV mass >20% of total LV mass) were fulfilled (8, 9). The presence of other LVNC related clinical features described by others or positive family history was not part of the inclusion criteria (10, 11). Patients with a reduced LV ejection fraction (EF; <50%); ischemic, valvular, or other myocardial or congenital heart disease; or other significant comorbidities (e.g., diabetes, hypertension, chronic kidney disease, or chronic liver failure) and patients whose CMR short-axis and long-axis cine images contained artifacts or were performed after the injection of contrast agent were excluded (12, 13). Clinical features of LVNC, including data about the patients' family history (cardiomyopathies, sudden cardiac death or congenital cardiac abnormalities), symptoms (e.g., syncope, palpitation, atypical chest pain), previous diagnosis of arrhythmia (supraventricular or ventricular extrasystoles, atrioventricular reentry tachycardia, atrioventricular nodal reentry tachycardia, or ventricular tachycardia), previously described non-ischemic electrocardiography (ECG) abnormalities (depolarization and repolarization abnormalities), history of embolic events (stroke, transient ischemic attack, or pulmonary embolism) or sudden cardiac death, were collected retrospectively by reviewing the patients' medical records and the completed questionnaires (10, 11). Patients were divided into two subgroups by the amount of RV trabecular mass indexed to body surface area (RV-TMi): those who exceeded the upper limit of the age and sex-specific RV-TMi reference range were assigned to the LVNC phenotype with RV hypertrabeculation (RV-HT, *n* = 22) subgroup, and those who were within the reference range were assigned to the LVNC phenotype with normal RV trabeculation (RV-NT, *n* = 78) subgroup (Figure 1) (14). The value of RV-TMi of each participant was individually checked and compared to the age and sex-related reference values (14). These reference values were set up previously on a large healthy cohort (100 male and 100 female participants) free of known disease using CMR and a threshold-based segmentation method, further described in the “Image Acquisition and Analysis” section. The population was divided into four different age groups set up with equal numbers of males and females (14). The mean and standard deviation or median and 95% confidence interval values of the used RV-TMi age- and sex-specific reference ranges are as follows: male: 20–29 years = 19.9 ± 3.7 g/m^2^, 30–39 years = 19.3 ± 3.8 g/m^2^, 40–49 years = 21.8 ± 5.2 g/m^2^, 50–66 years = 19.5 ± 3.6 g/m^2^, female: 20–29 years = 17.1 ± 3.6 g/m^2^, 30–39 years = 14.9 ± 3.0 g/m^2^, 40–49 years = 13.9 (11.9, 19.3) g/m^2^, and 50–66 years = 14.3 ± 2.8 g/m^2^ (14).

**Table 1:** Baseline characteristics of the study population.

	**LVNC**	**Control**	***p***
Number of participants (male)	100 (58)	100 (58)	-
Age (years)	37.5 ± 14.9	37.7 ± 13.4	0.678
BMI (kg/m^2^)	25.3 ± 4.0	24.4 ± 3.9	0.077
LV-EDVi (ml/m^2^)	76.8 ± 14.6	66.5 ± 10.5	**<0.001**
LV-ESVi (ml/m^2^)	26.4 ± 7.4	21.0 ± 5.4	**<0.001**
LV-SVi (ml/m^2^)	50.4 ± 9.4	45.5 ± 7.3	**<0.001**
LV-EF (%)	65.8 ± 5.6	68.7 ± 5.3	**<0.001**
LV-TMi (g/m^2^)	26.5 ± 7.3	20.7 ± 4.5	**<0.001**
LV-CMi (g/m^2^)	50.2 ± 12.4	46.3 ± 8.5	0.052
LV-GLS (%)	−21.9 ± 3.0	−23.5 ± 2.6	**<0.001**
LV-GCS (%)	−29.6 ± 5.0	−34.6 ± 4.9	**<0.001**
RV-EDVi (ml/m^2^)	72.2 ± 15.1	65.5 ± 12.7	**0.001**
RV-ESVi (ml/m^2^)	27.3 ± 7.7	24.3 ± 5.9	**0.005**
RV-SVi (ml/m^2^)	45.0 ± 9.0	41.2 ± 8.2	**0.002**
RV-EF (%)	62.4 ± 6.2	63.0 ± 4.9	0.528
RV-TMi (g/m^2^)	21.4 ± 6.2	17.9 ± 4.3	**<0.001**
RV-CMi (g/m^2^)	15.0 ± 4.5	15.5 ± 3.3	0.102
RV-GLS (%)	−25.2 ± 4.3	−27.3 ± 4.4	**<0.001**
RV-FWS (%)	−29.1 ± 5.0	−29.3 ± 6.4	0.830
RV-SS (%)	−16.3 ± 4.8	−19.1 ± 6.2	**<0.001**

*Bold values indicate statistical significance (p < 0.05)*.

*BMI, body mass index; CMi, compacted myocardial mass index; EDVi, end-diastolic volume index; EF, ejection fraction; ESVi, end-systolic volume index; FWS, free-wall strain; GCS, global circumferential strain; GLS, global longitudinal strain; LV, left ventricle; LVNC, left ventricular non-compaction; RV, right ventricle; SS, septal strain; SVi, stroke volume index; TMi, trabeculated myocardial mass index*.

Age- and sex-matched healthy volunteers who were free of known cardiovascular or other systemic diseases and who did not take medication were selected from our database as the control group. The average sports activity for both the patients and controls was <6 h/week (15). All procedures in this study were performed in accordance with the 1964 Helsinki Declaration and its later amendments or comparable ethical standards. Ethical approval was obtained from the Central Ethics Committee of Hungary, and all participants provided informed consent.

### Image Acquisition and Analysis

CMR examinations were performed on a 1.5 T MRI machine (Achieva, Philips Medical System, Eindhoven, the Netherlands). Balanced steady-state free precession cine images were obtained in 2-, 3-, and 4-chamber long-axis views and breath-hold short-axis views from base to apex with full coverage of the LV and RV before the administration of contrast agent (gadobutrol, 0.15 ml/kg). The scan parameters were as follows: repetition time, 2.7 ms; echo time, 1.3 ms; flip angle, 60°; spatial resolution, 1.5 × 1.5 mm; temporal resolution, 25 frames per cardiac cycle; slice thickness, 8 mm without interslice gap; and field of view, 350 mm on average adapted to body size.

Segmentation analysis was performed with Medis Suite QMass version 3.0 software (Medis Medical Imaging Systems, Leiden, the Netherlands). Automatic tracing with manual correction of the LV and RV endo- and epicardial contours was performed by one observer as described previously (16). Inter-and intraobserver agreement values were good-to-excellent and are presented in Supplementary Table 1. The MassK module of the QMass software was used to calculate the LV and RV volumetric and functional values and the myocardial mass values. This is a threshold-based papillary and trabeculated muscle quantification analysis software module that differentiates myocardial trabeculation from the blood pool based on differences in the signal intensity (16). Each voxel was classified as either blood or myocardium according to the chosen threshold (50%). Manual correction of the threshold was not applied. Papillary muscles and ventricular trabeculation were included in the endocardial contours and defined as trabeculated myocardial mass, while compacted myocardial mass was calculated as the difference between the total detected and trabeculated myocardial mass. LV and RV end-diastolic volume (LV-EDV, RV-EDV), end-systolic volume (LV-ESV, RV-ESV), stroke volume (LV-SV, RV-SV), ejection fraction (LV-EF, RV-EF), end-diastolic compacted myocardial mass (LV-CM, RV-CM), and end-diastolic trabeculated myocardial mass (LV-TM, RV-TM) were measured. The parameters were indexed (i) to body surface area using the Mosteller formula.

The QStrain module of Medis Suite version 3.0 was used for the feature-tracking strain analysis (Medis Medical Imaging Systems, Leiden, the Netherlands). To assess subendocardial strain, the endocardial contours were drawn in end-diastole and end-systole in the 2-, 3-, and 4-chamber long-axis and short-axis views of the LV and in the 4-chamber view of the RV, including endocardial trabeculation. The LV global longitudinal strain (LV-GLS), LV global circumferential strain (LV-GCS), RV-GLS, RV free-wall strain (RV-FWS), and RV septal strain (RV-SS) were measured.

### Statistical Analysis

Continuous parameters are described as the mean and standard deviation (SD), and discrete parameters are expressed as percentages. The intra-and interobserver agreement of the two observers was tested using the intraclass correlation coefficient (ICC). Distribution normality was assessed with the Shapiro–Wilk test. An unpaired Student's *t*-test or the Mann–Whitney test was used to compare the studied groups. Differences in normally distributed, variables with equal variance between the control and patient subgroups were analyzed with one-way analysis of variance (ANOVA) and Tukey's *post-hoc* test, while the Welch test and Games-Howell *post-hoc* test were used for variables with unequal variance; all other data were compared with the Kruskal–Wallis test with Bonferroni correction for multiple comparisons. Pearson's or Spearman's correlation was performed to describe the linear relationship between the parameters. The chi-squared test was used to compare discrete data. A *p*-value < 0.05 was considered indicative of statistical significance. IBM SPSS Statistics (Version 25.0, Armonk, NY) was used for calculations.

## Results

The baseline LV characteristics of the studied patients with the LVNC phenotype and the control subjects were compared (Table 1). Patients had significantly higher values of LV-EDVi, LV-ESVi, LV-SVi, and LV-TMi and significantly lower LV-EF, LV-GLS, and LV-GCS values (i.e., less negative strain values).

The comparison of the RV parameters yielded similar results: the RV-EDVi, RV-ESVi, RV-SVi, and RV-TMi values were significantly larger, and the RV-GLS and RV-SS values were significantly lower in the patient group, while the RV-EF, RV-CMi, and RV-FWS values were similar between the groups. Sixty-nine patients received contrast agent, but none demonstrated late gadolinium enhancement. Wall motion abnormalities were not visible in either the LV or RV in any patients.

**Figure 2:**
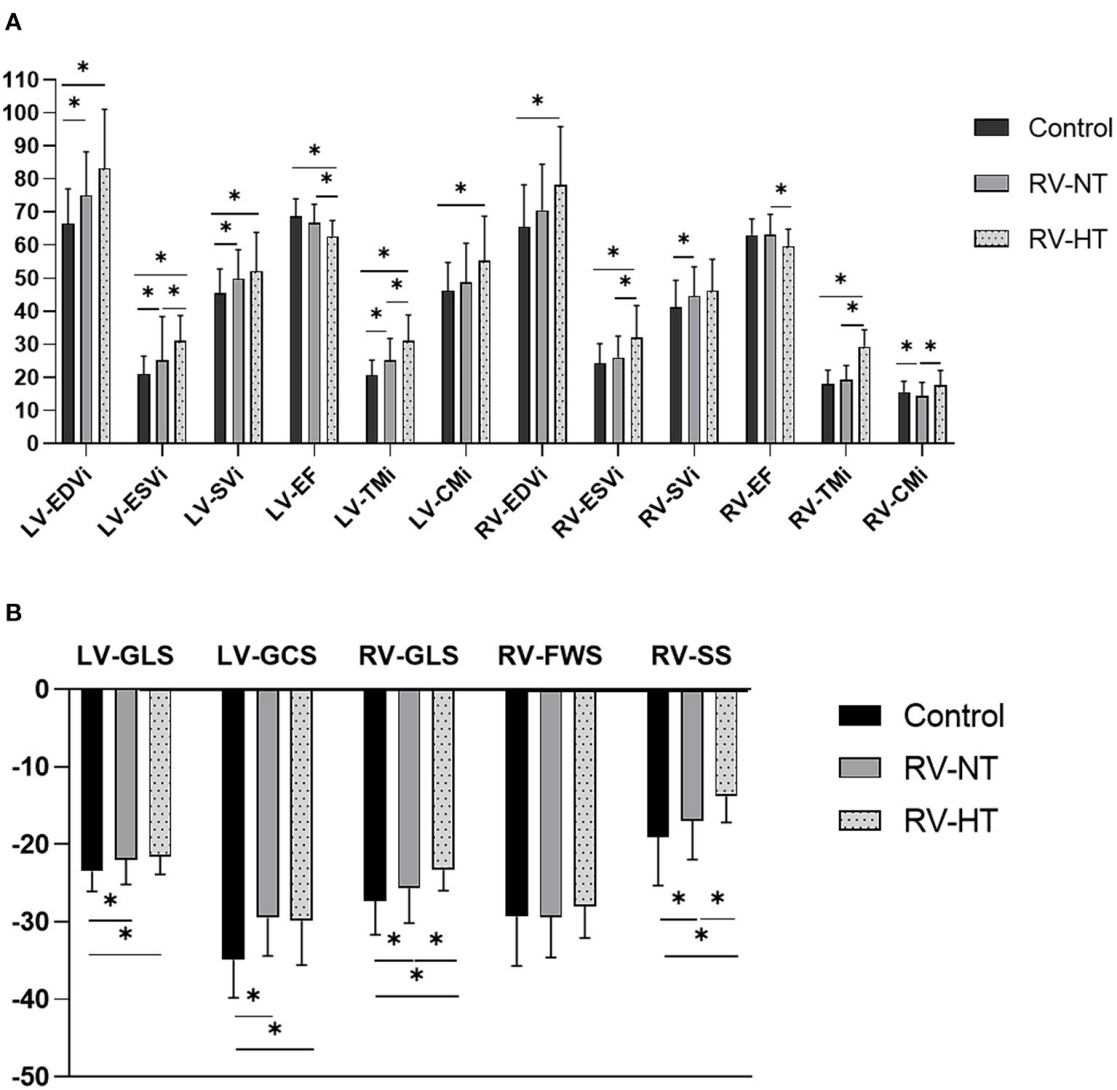
Graphic representation of Table 2. Comparison of the left and right ventricular volumetric and functional **(A)** and strain **(B)** parameters of LVNC patients with right ventricular hypertrabeculation, patients with normal right ventricular trabeculation, and controls. CMi, compacted myocardial mass index; EDVi, end-diastolic volume index; EF, ejection fraction; ESVi, end-systolic volume index; FWS, free-wall strain; GCS, global circumferential strain; GLS, global longitudinal strain; LV, left ventricle; LVNC, left ventricular non-compaction; RV, right ventricle; RV-HT, patients with right ventricular hypertrabeculation; RV-NT, patients with normal right ventricular trabeculation; SS, septal strain; SVi, stroke volume index; TMi, trabeculated myocardial mass index. *p < 0.05.

Next, the patients were divided into two subgroups by the amount of RV-TMi. Twenty-two patients had RV-TMi values that were higher than the age- and sex-specific reference values, forming the RV-HT subgroup (male: *n* = 15, mean age: 36.8 ± 3.8 years), while those whose RV-TMi values were within the normal reference range formed the RV-NT subgroup (*n* = 78, male: *n* = 43, mean age: 37.7 ± 1.6 years). None of the healthy control participants exceeded the upper normal limit for RV-TMi. We found significant differences between the two subgroups and the control group: the LV-ESVi, LV-TMi, RV-ESVi, RV-TMi, and RV-CMi values were significantly larger in the RV-HT group than in the RV-NT and control groups, the LV-EF, RV-GLS, and RV-SS values were significantly lower in the RV-HT group than in the RV-NT and control groups, and RV-EF values were significantly lower in the RV-HT group than in the RV-NT group (Table 2, Figure 2).

**Table 2:** Comparison of the left and right ventricular volumetric and functional (A) and strain (B) parameters of LVNC patients with right ventricular hypertrabeculation, patients with normal right ventricular trabeculation, and controls.

	**Control**	**RV-NT**	**RV-HT**	***p***
Age (years)	37.7 ± 13.4	37.7 ± 14.1	36.8 ± 17.9	**0.733**
LV-EDVi (ml/m^2^)	66.5 ± 10.5^¶^^#^	75.0 ± 13.2	83.2 ± 17.9	**<0.001**
LV-ESVi (ml/m^2^)	21.0 ± 5.4^¶^^#^	25.2 ± 6.9^#^^†^	31.0 ± 7.7^¶^^†^	**<0.001**
LV-SVi (ml/m^2^)	45.5 ± 7.3^¶^^#^	49.9 ± 8.7	52.2 ± 11.6	**<0.001**
LV-EF (%)	68.7 ± 5.3^¶^^#^	66.7 ± 5.6	62.7 ± 4.7	**<0.001**
LV-TMi (g/m^2^)	20.7 ± 4.5^¶^^#^	25.2 ± 6.6^#^^†^	31.0 ± 7.9^¶^^†^	**<0.001**
LV-CMi (g/m^2^)	46.3 ± 8.5^#^	48.7 ± 11.8	55.3 ± 13.4	**0.013**
LV-GLS (%)	−23.5 ± 2.6^¶^^#^	−22.0 ± 3.2	−21.6 ± 2.3	**<0.001**
LV-GCS (%)	−34.9 ± 4.9^¶^^#^	−29.5 ± 4.9	−29.9 ± 5.7	**<0.001**
RV-EDVi (ml/m^2^)	65.5 ± 12.7^#^	70.5 ± 14.0	78.2 ± 17.7	**<0.001**
RV-ESVi (ml/m^2^)	24.3 ± 5.9^¶^^#^	26.0 ± 6.5	32.0 ± 9.8	**<0.001**
RV-SVi (ml/m^2^)	41.2 ± 8.2^¶^	44.6 ± 8.9	46.3 ± 9.5	**0.007**
RV-EF (%)	63.0 ± 4.9	63.1 ± 6.2^#^	59.6 ± 5.2^¶^	**0.023**
RV-TMi (g/m^2^)	17.9 ± 4.3^#^	19.2 ± 4.4^#^	29.3 ± 5.1^¶^^†^	**<0.001**
RV-CMi (g/m^2^)	15.5 ± 3.3^#^	14.3 ± 4.2^#^^†^	17.6 ± 4.5^¶^	**0.001**
RV-GLS (%)	−27.3 ± 4.4^¶^^#^	−25.7 ± 4.5^#^^†^	−23.3 ± 2.7^¶^^†^	**<0.001**
RV-FWS (%)	−29.3 ± 6.4	−29.4 ± 5.2	−28.0 ± 4.1	0.608
RV-SS (%)	−19.1 ± 6.2^¶^^#^	−17.0 ± 5.0^#^^†^	−13.8 ± 3.4^¶^^†^	**<0.001**

*Bold values indicate statistical significance*.

*CMi, compacted myocardial mass index; EDVi, end-diastolic volume index; EF, ejection fraction; ESVi, end-systolic volume index; FWS, free-wall strain; GCS, global circumferential strain; GLS, global longitudinal strain; LV, left ventricle; LVNC, left ventricular non-compaction; RV, right ventricle; RV-HT, patients with right ventricular hypertrabeculation; RV-NT, patients with normal right ventricular trabeculation; SS, septal strain; SVi, stroke volume index; TMi, trabeculated myocardial mass index*.

¶ *p < 0.05 vs. RV-NT*.

# *p < 0.05 vs. RV-HT*.

† *p < . vs. Control*.

The linear relationship between RV-TMi, LV-TMi, and RV volumetric and functional parameters and left and right ventricular strain values were studied in the patient population (Table 3). A significant positive correlation was found between RV-TMi and LV-TMi, between RV-TMi and RV volumetric parameters, and between LV-TMi and RV volumetric parameters, while a larger RV-TMi value was associated with a lower RV-EF and worse RV-GLS, RV-SS, RV-FWS, and LV-GLS values. Furthermore, a higher LV trabecular mass was associated with worse RV-EF, RV-GLS, LV-GLS, and LV-GCS values.

**Table 3:** Correlation between right and left ventricular trabecular mass index and right ventricular parameters and left ventricular global strains.

	**RV-TMi**		**LV-TMi**	
	***r***	***p***	***r***	***p***
RV-EDVi	0.54	**<0.001**	0.48	**<0.001**
RV-ESVi	0.66	**<0.001**	0.53	**<0.001**
RV-SVi	0.34	**0.001**	0.34	**0.001**
RV-EF	−0.49	**<0.001**	−0.30	**0.002**
RV-TMi	1	0	0.59	**<0.001**
RV-CMi	0.69	**<0.001**	0.44	**<0.001**
RV-GLS	0.37	**<0.001**	0.22	**0.035**
RV-FWS	0.25	**0.014**	0.09	0.383
RV-SS	0.21	**0.038**	0.10	0.314
LV-GLS	0.24	**0.018**	0.38	**<0.001**
LV-GCS	0.03	0.809	0.28	**0.006**

*Bold values indicate statistical significance (p < 0.05)*.

*Pearson's or Spearman's correlation was performed to describe the linear relationship between the parameters. CMi, compacted myocardial mass index; EDVi, end-diastolic volume index; EF, ejection fraction; ESVi, end-systolic volume index; FWS, free wall strain; GCS, global circumferential strain; GLS, global longitudinal strain; LV, left ventricle; LVNC, left ventricular non-compaction; r, correlation coefficient; RV, right ventricle; SS, septal strain; SVi, stroke volume index; TMi, trabeculated myocardial mass index*.

We studied the frequency of the clinical features and family history of LVNC in the patients with the LVNC phenotype. Patients with multiple findings were found in each category (Table 4). Sixty-eight percent of patients had at least one clinical feature of LVNC. Documented arrhythmia was present in 22%, while 13% of the patients had palpitations without documented arrhythmia. Almost one-quarter (23%) of the patients had a positive family history, and in 10% of these patients, at least one other clinical feature of LVNC was present. Atypical chest pain was described in 11 cases, non-ischemic ECG abnormalities in nine patients, syncope in eight patients, thromboembolic events in three patients, and a non-fatal episode of cardiac arrest in one patient. No significant difference in the frequency of clinical features was found between the RV-HT and RV-NT subgroups.

**Table 4:** Prevalence of clinical features in the total patient population and patient subgroups.

**A**.	**Clinical features**	**LVNC total (*n* = 100)**	**RV-HT (*n* = 22)**	**RV-NT (*n* = 78)**	**RV-HT vs. RV-NT *p***
	Palpitation	20%	23%	19%	0.717
	Palpitation with documented arrhythmia	7%	9%	6%	0.663
	Palpitation without documented arrhythmia	13%	14%	13%	0.920
	Arrhythmia	22%	27%	21%	0.499
	Non-sustained ventricular arrhythmia	3%	5%	3%	0.630
	Ventricular extrasystoles	15%	14%	15%	0.839
	Supraventricular extrasystoles	4%	9%	3%	0.168
	Atrioventricular reentry tachycardia	2%	5%	1%	0.334
	Bradycardia	1%	5%	0%	0.058
	Syncope	8%	14%	6%	0.270
	Non-ischemic ECG abnormalities	9%	5%	10%	0.408
	Previous thromboembolic event	3%	5%	3%	0.630
	Transient ischemic attack	1%	0%	1%	0.594
	Stroke	1%	5%	0%	0.058
	Pulmonary microembolism	1%	0%	1%	0.594
	Sudden cardiac death	1%	0%	1%	0.594
	Positive family history	23%	18%	24%	0.543
	Positive family history and other clinical features	10%	14%	9%	0.520
	Atypical chest pain	11%	9%	12%	0.746
	No symptoms or family history	32%	32%	32%	0.983

*LVNC, left ventricular non-compaction; RV-HT, patients with right ventricular hypertrabeculation; RV-NT, patients with normal right ventricular trabeculation*.

We also compared the LV and RV parameters of the patients with LVNC phenotype with at least one clinical feature (*n* = 68) and without any clinical features (*n* = 32). The LV-CMi and RV-ESVi values were significantly lower in the patient with at least one clinical feature subgroup. However, all of the other measured RV and LV parameters were comparable between the groups (Table 5).

**Table 5:** Comparison of the left and right ventricular parameters of the patients with LVNC phenotype and at least one clinical feature and the patients with LVNC phenotype and no clinical features.

	**Patients with LVNC phenotype and**	**Patients with LVNC phenotype and**
	**at least one clinical feature (*n* = 68)**	**no clinical features (*n* = 32)**	***p***
Age (years)	34.8 ± 12.3	38.8 ± 16.0	0.212
LV-EDVi (ml/m^2^)	75.1 ± 14.5	80.3 ± 14.5	0.094
LV-ESVi (ml/m^2^)	25.7 ± 7.9	28.0 ± 6.2	0.152
LV-SVi (ml/m^2^)	49.5 ± 9.2	52.3 ± 9.7	0.164
LV-EF (%)	66.1 ± 6.2	65.5 ± 4.2	0.455
LV-TMi (g/m^2^)	26.1 ± 7.6	27.2 ± 6.8	0.504
LV-CMi (g/m^2^)	48.2 ± 11.7	54.4 ± 13.0	**0.019**
LV-GLS (%)	−22.3 ± 3.0	−21.1 ± 3.0	0.051
LV-GCS (%)	−30.1 ± 5.2	−28.6 ± 4.6	0.163
RV-EDVi (ml/m^2^)	70.2 ± 15.1	76.5 ± 14.5	0.052
RV-ESVi (ml/m^2^)	26.1 ± 7.9	29.9 ± 6.8	**0.022**
RV-SVi (ml/m^2^)	44.2 ± 9.1	46.6 ± 8.8	0.211
RV-EF (%)	63.0 ± 6.9	61.0 ± 4.2	0.146
RV-TMi (g/m^2^)	20.8 ± 6.0	22.9 ± 6.5	0.111
RV-CMi (g/m^2^)	14.5 ± 4.5	16.2 ± 4.3	0.067
RV-GLS (%)	−25.5 ± 4.0	−24.4 ± 4.8	0.23
RV-FWS (%)	−29.7 ± 4.8	−27.7 ± 5.2	0.059
RV-SS (%)	−16.0 ± 4.4	−17.0 ± 5.7	0.344

*Bold values indicate statistical significance (p < 0.05)*.

*CMi, compacted myocardial mass index; EDVi, end-diastolic volume index; EF, ejection fraction; ESVi, end-systolic volume index; FWS, free wall strain; GCS, global circumferential strain; GLS, global longitudinal strain; LV, left ventricle; LVNC, left ventricular non-compaction; RV, right ventricle; SS, septal strain; SVi, stroke volume index; TMi, trabeculated myocardial mass index*.

## Discussion

This study described the RV volumetric, functional, and strain characteristics and clinical features of patients with LVNC phenotype and good LV function using CMR. We also identified differences between LVNC phenotype patients with RV hypertrabeculation and those with normal RV trabeculation.

We observed higher LV and RV volumes, lower LV-EF, and lower LV and RV strain values in LVNC phenotype patients than in controls. These might be related to excessive trabeculation, but the clinical relevance is controversial. We also need to mention that all of these parameters were in the normal range. Kawel et al. found that more prominent LV trabeculation was associated with lower LV-EF and higher LV volumes in a population with no known cardiovascular disease or diagnosed LVNC but presenting with LV hypertrabeculation (4). A geometric model might address the physiologic explanation for these observations: a ventricle with trabeculation can maintain the SV with less deformation than a smooth-walled ventricle, which needs to generate much more deformation to keep the SV (17). However, trabeculation occupies space of its own. Thus, the ventricle needs to be dilated, suggesting that the presence of excessive RV trabeculation causes increased RV volumes (17). In our study, almost one-quarter of the patients with LVNC phenotype had RV trabecular mass that exceeded the upper value of the age and sex-specific reference range (14). These patients had higher LV and RV volumetric values, lower RV and LV-EF, and worse RV-GLS and RV-SS values than patients whose RV trabecular mass values were within the reference range. The positive correlation between RV volumetric parameters and RV trabecular mass, and the inverse relationship between RV trabecular mass and RV strain values further strengthen the hypothesis that excessive RV trabeculation might be the cause of these alterations. According to these results, the volumetric and functional characteristics in a hypertrabeculated RV are similar to those in LV non-compaction, suggesting RV non-compaction in some patients with LVNC.

However, the clinical importance of these findings and RV hypertrabeculation in the setting of good LV and RV function are difficult to evaluate due to the lack of follow-up studies in this patient population. One study found no relation between the presence of RV non-compaction and RV dysfunction; furthermore, LV-EF was the only independent predictor of RV-EF in patients with LVNC (18). The most common cause of RV dysfunction is LV dysfunction in non-ischemic cardiomyopathy due to several factors, including ventricular interdependence with septal dysfunction (19). The ventricular septum accounts for the major force of RV ejection due to its twisting and shortening during systole, which is caused by the oblique fiber orientation of the septum (20). In the case of ventricular dilatation, the oblique fibers of the septum become more transverse, resulting in a decrease in septal twisting and shortening and causing decreased RV ejection (20, 21). We described an association between a higher LV trabecular mass value and a higher RV trabecular mass, higher RV volumes, lower RV-EF, and a worse RV-GLS value, which might strengthen the hypothesis that LV involvement affects RV function. Furthermore, we found that a higher RV-TMi value was associated with a worse RV-SS value. It supports the theory that RV dysfunction arises from septal dysfunction, both of which can be observed even in cases of good LV function in patients with LVNC phenotype.

Previous studies have suggested that myocardial mass, including ventricular trabeculation, is genetically determined (22, 23). The strong relationship between LV and RV trabecular mass further indicates that the genetic mutations underlying LV non-compaction might also affect RV trabeculation, causing RV non-compaction in some cases.

None of the healthy control participants exceeded the upper limit of the age and sex-specific reference values in this study. Stämpfli et al. studied the RV end-diastolic trabeculated area and trabeculated volume in LVNC patients and controls (24). However, they described a significant overlap in RV trabeculation of patients and controls, which several factors might cause. First, they used manual contouring to measure RV trabeculated volume, which is different from ours. Second, they did not use age and sex-specific normal reference ranges to differentiate between normal and excessive RV trabeculation. Third, the number of included participants was smaller in their study compared to ours. Although, an overlap cannot be excluded in the case of an even larger study population.

When assessing the clinical features of the patients with the LVNC phenotype, we found that two-thirds had at least one risk factor requiring regular follow-up and that these patients might be diagnosed with true LVNC (10, 11). The prevalence of positive family history and arrhythmias is similar to that in previous reports; however, chest pain, syncope, and non-ischemic ECG abnormalities were less common in our population (10, 25, 26). Previous studies describing the clinical features of LVNC involved patients who presented to the corresponding study centers due to their symptoms and decreased LV function. In contrast, we included LVNC phenotype patients with good LV ejection fraction; thus, the lower prevalence of clinical findings in our study might be due to differences in the patient populations.

Patients with the LVNC phenotype and at least one clinical feature had a significantly lower LV-CMi value than patients without clinical features. Gebhard et al. described the thinning of the compacted myocardial layer in patients with LVNC using echocardiography (27). Our results might support this criterion; however, it is interesting that no other difference was found between the with and without clinical features subgroups.

One-third of our study's total LVNC phenotype patient population had a negative family history and no symptoms; this prevalence is consistent with the literature (28). According to current knowledge, this “benign form” of LVNC has a good prognosis, and patient follow-up is not necessary (11, 29).

Case reports and previous studies have described the presence of complex ventricular extrasystoles, ventricular tachycardia, and sudden cardiac death in patients with RV non-compaction and some authors have suggested that RV non-compaction is an arrhythmogenic state associated with high mortality (1, 2, 30). However, these patients had either RV dysfunction or congenital cardiac abnormalities that affected the right heart. Furthermore, our study showed that the prevalence of clinical features was similar between patients with RV hypertrabeculation and patients with normal RV trabeculation. Further follow-up studies are necessary to evaluate RV function, clinical features, and major cardiovascular events in the presence of RV hypertrabeculation/non-compaction in patients with LVNC.

To conclude, this study found that almost one-quarter of the patients with the LVNC phenotype had RV trabecular mass values higher than the age and sex-specific reference range. Furthermore, the volumetric, functional, and strain characteristics of the hypertrabeculated RV were similar to these parameters of the LV in LVNC. We described a positive correlation between RV and LV trabeculation, RV trabeculation and RV volumes, and significant inverse relationships between RV and LV trabeculation with RV function. These results suggest that a portion of patients with the LVNC phenotype might also have RV non-compaction, raising further questions about its long-term effect on RV function, clinical manifestation, and patient prognosis. Further follow-up studies are necessary to answer these questions.

We need to mention that the study's main limitations arise from its retrospective nature and the lack of follow-up information on the patients. We also have to mention the limitations of the threshold-based software. The software currently quantifies ejection fraction and volume using short-axis slices, with an 8 mm standard for spatial resolution in the Z-direction. Trabeculae and the papillary muscles do not cross the slice perfectly perpendicularly, resulting in partial volume effects. Depending on the actual path of the trabeculae, this influences the threshold-based quantification (16, 31). The limitations of the feature-tracking software include the lack of a relevant validation process; thus, its clinical application is questionable. Furthermore, there is high variability in normal strain values between different vendors; thus, there is a lack of an accepted normal reference range (32, 33).

## Data Availability Statement

The raw data supporting the conclusions of this article will be made available by the authors, without undue reservation.

## Ethics Statement

The studies involving human participants were reviewed and approved by Central Ethics Committee of Hungary. The patients/participants provided their written informed consent to participate in this study.

## Author Contributions

ARK: methodology, formal analysis, investigation, resources, data curation, writing—original draft, and visualization. ZG and AP: data curation. LS, ZD, AK, and BL: writing—review and editing. BM: supervision. HV: writing—review and editing and supervision. AS: conceptualization, methodology, investigation, resources, writing—review and editing, supervision, and project administration. All authors contributed to the article and approved the submitted version.

## Funding

The research was financed by the Thematic Excellence Programme (Tématerületi Kiválósági Program, 2020-4.1.1.-TKP2020) of the Ministry for Innovation and Technology in Hungary within the framework of the Therapeutic Development and Bioimaging Programs of Semmelweis University; by the Development of Scientific Workshops of Medical, Health Sciences and Pharmaceutical Education (Project identification number: EFOP-3.6.3-VEKOP-16-2017-00009); and by the Ministry of Innovation and Technology NRDI Office within the framework of the Artificial Intelligence National Laboratory Program. Project no. NVKP_16-1–2016-0017 (National Heart Program) has been implemented with the support provided by the National Research, Development, and Innovation Fund of Hungary, financed under the NVKP_16 funding scheme.

## Conflict of Interest

The authors declare that the research was conducted in the absence of any commercial or financial relationships that could be construed as a potential conflict of interest.

## Publisher's Note

All claims expressed in this article are solely those of the authors and do not necessarily represent those of their affiliated organizations, or those of the publisher, the editors and the reviewers. Any product that may be evaluated in this article, or claim that may be made by its manufacturer, is not guaranteed or endorsed by the publisher.
