# Supplementary material for: Impact of Right Ventricular Trabeculation on Right Ventricular Function in Patients With Left Ventricular Non-compaction Phenotype
Source: Front Cardiovasc Med. 2022 Apr 12;9:843952. doi: 10.3389/fcvm.2022.843952 (PMC9041027; doi:10.3389/fcvm.2022.843952)
Supplement: Supplementary file 1 [file Table_1.pdf]

Supplementary Table 1. Intraclass correlation coefficient (ICC) of the measured left and right ventricular parameters to describe inter-and intraobserver agreement. An intraclass correlation coefficient (ICC) less than 0.4, between 0.4 and 0.75, and greater than 0.75 indicates poor, fair to good, and excellent interobserver agreement.

CMi: compacted myocardial mass index, EDVi: end-diastolic volume index, EF: ejection fraction, ESVi: end-systolic volume index, FWS: free wall strain, GCS: global circumferential strain, GLS: global longitudinal strain, LV: left ventricle, LVNC: left ventricular noncompaction, RV: right ventricle, SS: septal strain, SVi: stroke volume index, TMi: trabeculated myocardial mass index

	**Interobserver agreement (ICC)**	**Intraobserver agreement (ICC)**
**LV-EDVi**	0.98 (0.86-0.99)	0.99 (0.99-0.99)
**LV-ESVi**	0.96 (0.90-0.98)	0.95 (0.90-0.98)
**LV-SVi**	0.95 (0.85-0.98)	0.99 (0.98-0.99)
**LV-EF**	0.95 (0.89-0.98)	0.98 (0.95-0.99)
**LV-TMi**	0.99 (0.95-0.99)	0.98 (0.95-0.99)
**LV-CMi**	0.99 (0.98-0.99)	0.99 (0.98-0.99)
**LV-GLS**	0.96 (0.89-0.98)	0.97 (0.92-0.99)
**LV-GCS**	0.96 (0.89-0.98)	0.94 (0.87-0.97)
**RV-EDVi**	0.99 (0.97-0.99)	0.99 (0.98-0.99)
**RV-ESVi**	0.96 (0.93-0.99)	0.95 (0.87-0.97)
**RV-SVi**	0.99 (0.97-0.99)	0.99 (0.98-0.99)
**RV-EF**	0.98 (0.95-0.99)	0.92 (0.82-0.97)
**RV-CMi**	0.98 (0.95-0.99)	0.83 (0.62-0.92)
**RV-TMi**	0.99 (0.97-0.99)	0.98 (0.96-0.99)
**RV-GLS**	0.83 (0.63-0.93)	0.92 (0.83-0.97)
**RV-FWS**	0.75 (0.43-0.89)	0.83 (0.78-0.95)
**RV-SS**	0.75 (0.44-0.89)	0.83 (0.17-0.94)
